# Case of a Singular Cutaneous Affection; with Some Remarks Relative to the Poison of Copper

**Published:** 1792

**Authors:** William Davidson

**Affiliations:** Apothecary in London.


IX. *Cafe of a fingular cutaneous Affection; with fome Remarks relative to the Poifon of Copper. By Mr.* William Davidfon, *Apothecary in London. Communicated in a Letter to Dr.* Seguin Henry Jackfon, *Phyfician in London, and by him to Dr.* Simmons.

ON the 16th day of December, 1787, I was fent for to a family in my neighbourhood, where the mother and four children were affected with a cutaneous eruption, which had made its appearance that day, and occafioned them much uneafinefs.

uneaſineſs. This eruption was ſeemingly of the leprous kind, and conſiſted of ſpots of different ſizes, the largeſt of which were white and ſcaly, with moiſt baſes, appearing as if ſomething acrimonious had been ſecreted under the cuticle, which thickened, raiſed, and ſeparated it from the cutis. It was more or leſs all over the body, and very much amongſt the hair of the head. There was no itching, or particular pain; nor did the general health of the patients ſeem affected.

Knowing that affections of the ſkin often ariſe from thoſe of the ſtomach, I queſtioned them about their diet, and particularly inquired whether they had taken any thing that day which had diſagreed with them. I was informed, that, two days before, the above five perſons and three young women (viſitors) had all dined upon peaſe ſoup, which was diſtributed with a braſs ladle that had been in the warm ſoup ſome little time; that the ladle, having been long out of uſe, and hung up expoſed to the open air, was quite green, but which was not noticed at the time; and that the mother, having been much alarmed, and imputing the appearances on the ſkin to this circumſtance, had ſent to the three viſitors, and, finding they were all affected in in the ſame manner, concluded they were poiſoned, unleſs I could afford them ſome immediate relief.

The mother was about twenty-ſix years of age, and in the ſecond month of her pregnancy, although ſhe was ſtill ſuckling the youngeſt of the patients, a girl then about fourteen months old. The ſpots upon this child were not very numerous; and it was obſerved that ſhe had taken only a ſmall quantity of the ſoup. The eldeſt daughter was between eleven and twelve years of age; the next, about nine; and the other child, about four. The young women were about ſeventeen or eighteen. None of them had taken any thing which had occaſioned ſickneſs or any internal affection; and even the peaſe ſoup, which certainly contained more or leſs of verdigris, had occaſioned no diſturbance in the ſtomach, and was digeſted as uſual.

I found, on examination, that the quantity of eruption was in an exact proportion to the quantity of ſoup taken by each perſon; for the mother, who wiſhed to avoid peaſe ſoup, thinking it improper for her who was then giving ſuck, and only took a ſmall quantity, had leſs of the eruption than any of the reſt; while one of the girls, who had eaten plentifully of it, was almoſt moſt covered with this leprous appearance. From this laſt circumſtance, and from the length of time which intervened between the taking of the ſoup and the appearance of the ſpots upon the ſkin, I was led to believe that this eruption could not originate from that ſympathy between the ſkin and ſtomach to which may be aſcribed ſo many of the cutaneous affections, but ought rather to be imputed to the copper mixed with the ſoup, and which, having been taken into the ſtomach, abſorbed, and carried into the blood veſſels, the conſtitution was now endeavouring to diſcharge by throwing it upon the ſurface of the body. I, therefore, conceived that the cure would chiefly depend upon encouraging theſe efforts, and at the ſame time, if poſſible, deſtroying the virulence of whatever portion of the copper might remain in the blood veſſels.

Having theſe conſiderations in view, and recollecting the well-known effects of ſulphur, not only as a diaphoretic, but alſo in rendering the moſt active metallic ſubſtances mild and innocent, I conſidered it as the beſt medicine I could employ. Accordingly I adminiſtered the *lac ſulphuris* night and morning, in doſes ſo regulated that its chief action might not be exerted erted in the ſtomach and bowels; fearing leſt, by an increaſed proportion, the periſtaltic motion might be ſo much excited as to check the determination to the ſurface of the body.

Had any urgent ſymptoms been preſent, I ſhould have given it every four or ſix hours. In all the patients the body continued ſufficiently open, and there was more or leſs of gentle perſpiration in the night. For the firſt five days the ſpots kept coming out; but from that period they became browner, and at laſt gradually diſappeared, without leaving any mark behind. The medicine was continued only about ſeven or eight days, as the patients felt themſelves in perfect health, and the eruption ſeemed going off. Indeed the whole ſoon diſappeared, excepting on the girl who had eaten moſt heartily of the ſoup. Upon her it continued for near two months. The family remained afterwards in perfect health, and have never had any return of this or any ſimilar complaint; nor were they ever before affected with any diſeaſe of this kind.

I ſhall juſt beg leave to obſerve, that the ſoup was made of freſh beef, and in a tin ſaucepan; ſo that, excepting the verdigris on the ladle, there was no other ſubſtance which could produce any of the above-mentioned appearances*. Here the copper was mixed in a vehicle the beſt adapted for leſſening its ſtimulating powers, for blunting its emetic property, and allowing it inſidiouſly to get into the blood. Whether it there retained its original form of aërated or acetated copper, and was in that ſtate effuſed under the cuticle by the exhalent arteries, ſome future experiments may, perhaps, determine. It is well known that copper, in this ſtate, will diſſolve in a watery menſtruum; for a conſiderable portion of verdigris will diſſolve in cold diſtilled water, making a green ſolution.

If then my inference be allowed, that the copper, a very irritating, poiſonous ſubſtance, was ſo ſheathed as to be introduced into the ſyſtem without producing any particular effects in the *primæ viæ*, might not ſome of our more active medicines be in like manner conveyed into the blood veſſels, ſo as to excite actions in the more remote parts of our ſanguiferous and glandular ſyſtems, which might tend to the cure of the more obſtinate diſeaſes? Every practitioner knows that mercury may be ſpeedily

* Is it neceſſary for me to inform the chemical reader that the ſoup was ſeaſoned with common ſalt?

introintroduced into the blood in this way, without paſſing off by the bowels. But at ſome future period chemiſtry may, perhaps, furniſh us with ſome medicines ſtill more active and effectual, which, introduced in this way, may produce ſpecific actions tending to the reſtoration of health.

It may perhaps be worthy of inquiry, whether verdigris produced from pure copper, or from that metal when in the form of braſs, produces ſimilar effects in the human body? A circumſtance having lately occurred which has ſome relation to this ſubject, I ſhall beg leave to mention it:—About four or five weeks ago a fine little boy was brought to me, having upon him a leprous eruption, which appeared ſimilar to the above. He was about three years of age, of a florid complexion, lively, and ſeemingly in good health. The eruption had been out about ſeven or eight days. Having in remembrance the caſe juſt now related, I enquired whether the boy had been in the habit of putting any thing made of copper or braſs into his mouth? His mother told me that he did ſo very often; that he uſed frequently to put halfpence and a braſs thimble into his mouth, which (ſhe ſaid) occaſioned neither ſoreneſs in the mouth, nor ſicknefs of ftomach. From this account I imagined that the eruption might arife from the fame caufe as in the preceding cafes, (viz. from copper, abforbed, carried into the blood, and thrown out upon the fkin) and therefore determined to try the fame remedy. I accordingly ordered eight powders for him, each confifting of ten grains of the *lac fulphuris*; one of thefe was directed to be taken night and morning. His mother was defired to bring him to me again in four or five days; but as fhe omitted to do this, I am unable to give the event of the cafe.

*Queen Anne Street, Eaft,*

July 13th, 1792.

